# Modulation of long-term potentiation following microdoses of LSD captured by thalamo-cortical modelling in a randomised, controlled trial

**DOI:** 10.1186/s12868-024-00844-5

**Published:** 2024-02-05

**Authors:** Robin J. Murphy, Kate Godfrey, Alexander D. Shaw, Suresh Muthukumaraswamy, Rachael L. Sumner

**Affiliations:** 1 School of Pharmacy, University of Auckland, Auckland, New Zealand; 2 Centre for Psychedelic Research, Department of Psychiatry, Imperial College London, London, UK; 3 Department of Psychology, Exeter University, Exeter, UK

**Keywords:** Long-term potentiation, Psychedelics, Neuroplasticity, Lysergic acid diethylamide, Dynamic causal modelling

## Abstract

**Background:**

Microdosing psychedelics is a phenomenon with claimed cognitive benefits that are relatively untested clinically. Pre-clinically, psychedelics have demonstrated enhancing effects on neuroplasticity, which cannot be measured directly in humans, but may be indexed by non-invasive electroencephalography (EEG) paradigms. This study used a visual long-term potentiation (LTP) EEG paradigm to test the effects of microdosed lysergic acid diethylamide (LSD) on neural plasticity, both acutely while on the drug and cumulatively after microdosing every third day for six weeks. Healthy adult males (*n* = 80) completed the visual LTP paradigm at baseline, 2.5 h following a dose of 10 µg of LSD or inactive placebo, and 6 weeks later after taking 14 repeated microdoses. Visually induced LTP was used as indirect index of neural plasticity. Surface level event-related potential (ERPs) based analyses are presented alongside dynamic causal modelling of the source localised data using a generative thalamocortical model (TCM) of visual cortex to elucidate underlying synaptic circuitry.

**Results:**

Event-related potential (ERP) analyses of N1b and P2 components did not show evidence of changes in visually induced LTP by LSD either acutely or after 6 weeks of regular dosing. However modelling the complete timecourse of the ERP with the TCM demonstrated changes in laminar connectivity in primary visual cortex. This primarily included changes to self-gain and inhibitory input parameters acutely. Layer 2/3 to layer 5 excitatory connectivity was also different between LSD and placebo groups. After regular dosing only excitatory input from layer 2/3 into layer 5 and inhibitory input into layer 4 were different between groups.

**Conclusions:**

Without modulation of the ERPs it is difficult to relate the findings to other studies visually inducing LTP. It also indicates the classic peak analysis may not be sensitive enough to demonstrate evidence for changes in LTP plasticity in humans at such low doses. The TCM provides a more sensitive approach to assessing changes to plasticity as differences in plasticity mediated laminar connectivity were found between the LSD and placebo groups.

*Trial registration:* ANZCTR registration number ACTRN12621000436875; Registered 16/04/2021 https://www.anzctr.org.au/Trial/Registration/TrialReview.aspx?id=381476.

**Supplementary Information:**

The online version contains supplementary material available at 10.1186/s12868-024-00844-5.

## Background

Microdosing of psychedelics is an increasingly documented phenomenon [1], which has drawn substantial scientific interest in recent years,due to its claimed benefits to mental health and cognitive functioning [2]. The practise involves repeatedly taking doses of a psychedelic drug at quantities below the threshold for producing hallucinogenic effects [2, 3]. Recent work in our lab has demonstrated acute mood-elevating effects when administered in naturalistic environments in healthy adults, however the mechanism of these changes remains unclear and this effect has not yet been tested in clinical populations [4].

One proposed mechanism of microdosing’s reported effects is modulation of neuroplasticity [5–8]. Neuroplasticity refers to the reorganisation of neurons and their connections in response to experience, and is a crucial mechanism of learning, memory, and other adaptive processes in the central nervous system [9]. Impaired neuroplasticity has been theorised to contribute to the pathophysiology of depression [10], and to cognitive decline in ageing [11]. Serotonergic psychedelics including lysergic acid diethylamide (LSD) have been termed psychoplastogens [12], due to their ability to trigger structural and functional changes to neuroplasticity, directly measurable in preclinical models [13].

While in vitro and ex vivo studies of neural plasticity are not viable in human clinical trials, functional changes to plasticity may be able to be indexed via electroencephalography (EEG) paradigms and modelled in silico. Long term potentiation (LTP), characterized by the increased response of post-synaptic neurons following the tetanisation of pre-synaptic neurons with a high frequency stimulus, has been investigated as an index of Hebbian plasticity – in which learning and memory are a proposed function of the concurrent activation of pre- and post-synaptic neurons leading to increased synaptic efficacy [14]. LTP was first observed in an animal in vivo model, in which high frequency electrical stimulation (tetanisation) of a pre-synaptic neuron subsequently increased the excitation of a post-synaptic neuron, observable for many hours after the initial stimulation [15]. Following this, non-invasive visual evoked potential (VEP) LTP paradigms have been developed for use in humans, which are theorized to index these same effects non-invasively [14]. Critically, the paradigms induce an LTP-like effect with repeated visual stimulation and record the product of the induced changes as enhanced VEPs in response to the same stimuli. Of these, the Teyler protocol used in the current study [16], has been shown to produce VEP components which are modulated in relation to ageing [17–19], autism spectrum disorder [20], depression [21], and in combined oral contraceptive use [22]. Crucially, these changes have been associated with pro-plasticity genetic polymorphisms and shown to be predictive of memory task performance [23].In place of direct electrical stimulation, the Teyler protocol uses high-contrast visual stimuli that are administered as a high frequency photic tetanus, leading to observable changes to VEP components: the N1b (lateralized trailing edge of the negative N1 component occurring around 132–200ms) and P2 (centralized positivity occurring around 178-240ms) [14]. The N1b is typically altered in early post-tetanus recordings, and is sensitive to stimulus orientation, but modulation becomes less noticeable in late post-tetanus recordings. In contrast, P2 is typically modulated in late post-tetanus recordings and is not sensitive to orientation. These differences suggest that N1b and P2 may be indexing different mechanisms, likely short-term potentiation (STP), a rapid, but unstable precursor to more enduring LTP, and early LTP (e-LTP) respectively [14, 24].

LTP was first identified by direct stimulation of animal hippocampal tissue [15] and has been replicated in other animal cortical tissue [25] as well as human hippocampal tissue excised from people with epilepsy [26]. Beyond the visual paradigms, non-invasive modes of inducing LTP in humans, involving auditory [27], tactile [28], or transcranial magnetic stimulation [29] tetani have demonstrated its generalisability to other cortical regions, although none have been as consistently tested as visual paradigms [14]. Further to this, visual LTP demonstrated with the current paradigm has previously been correlated to visual memory performance [23], suggesting a relationship with higher-level learning and memory functions [30].

A previous limitation of this approach has been the inability to infer the mechanism underlying apparent increases or decreases in the VEP in response to photic-tetanus. EEG data primarily reflects the activity of superficial (layers 2/3) cortical cells (with contribution from layer 5 pyramidal cells) [31], with this behaviour modulated by hierarchically organised networks of inhibitory and excitatory connections from deeper cortical layers, as well as sub-cortical structures, particularly the thalamus [32]. While direct recording of these deeper cell populations is not viable in humans in a trial such as this, methods such as dynamic causal modelling (DCM) have been used to apply mathematical neural mass models of human brain architecture to neurophysiological data to gain insight into the activity of cell populations that are likely causing the effects observable at the scalp [33]. A biologically grounded model of thalamo-cortical circuitry [34] has previously been used to build a neural mass model of human brain networks related to visual processing, such that the production of signal at the scalp is plausibly explained by alterations to the connectivity between and within cell populations in the thalamus and layers 2–6 of the visual cortex, as well as receptor time constants (open time and decay rate) of neurotransmitters [35]. Application of this thalamo-cortical model (TCM) to EEG LTP data without a drug condition has previously demonstrated modulation of connections consistent with in vivo LTP research, including increased excitatory connectivity between the thalamus and layer 4 neurons in the visual cortex, as well as from layer 4 to layers 2/3 [36], but has not yet been applied to microdoses of LSD.

No visual LTP paradigm has yet been tested in humans following administration of serotonergic psychedelics in either low or full doses, however there have been some investigations with other drugs and paradigms. The Teyler protocol has been applied in the early post-acute phase of full doses of the non-serotonergic psychoplastogen ketamine [21]. Ketamine administration enhanced potentiation of the late P2 component in patients with Major Depressive Disorder (MDD), suggesting a pro-plastic effect. An auditory LTP task has been administered following a full dose of the serotonergic psychedelic psilocybin to MDD patients, and had no effect on 24 h after the dose, but showed a pro-plastic effect 2 weeks following the dose [37].

Previous administration of non-LTP EEG protocols under LSD microdoses have demonstrated changes to ERPs and oscillations [38, 39]. A study using psilocybin microdoses that were not laboratory-supplied also found alterations to resting oscillations but not ERPs following an oddball task [40]. Together this shows that even very low doses are able to occasion measurable changes to brain activity.

We administered a version of the Teyler visual LTP protocol [21] to healthy male participants in the acute phase of an LSD microdose and after six weeks of repeated microdosing, as part of a randomised controlled trial of at-home microdosing [4, 41]. Sustained effects were measured two days after the final microdose. To investigate the neural bases of these changes, we then applied a DCM TCM to the source reconstructed data [35, 36].

Much of the pre-clinical research on psychedelics and neural plasticity have focussed on lasting effects [5]. Therefore we hypothesized that LSD microdoses would increase potentiation of LTP-associated ERPs in the visual cortex, in particular late P2 and possibly the early N1b from baseline to the final EEG after six weeks of regular microdosing. We expected the TCM output would also demonstrate parameter modulation differences between placebo and LSD consistent with known electrophysiological and pharmacodynamic effects of psychedelics on enhancing plasticity [13].

The acute effects of LSD on plasticity related VEP modulation were exploratory as invasive and pre-clinical studies haven’t tested in this time frame. However, again the TCM output was expected to demonstrate parameter modulation differences between placebo and LSD consistent with known electrophysiological and pharmacodynamic effects of LSD. These may capture effects including LSD suppression of neural firing [42].

## Methods

This research was conducted at the University of Auckland from April 2021 to July 2022 as part of the MDLSD study described elsewhere [4, 41]. Healthy male volunteers (*n* = 80) were randomised into LSD or placebo groups in a 1:1 allocation ratio (see Additional file 1 for further description of randomisation and sample). Only male participants were recruited due to previously reported evidence that the LTP response varies significantly across the menstrual cycle and the unfeasibility of phase-locking measure points with a sample of this size [43]. Participants undertook the EEG protocol at three occasions: at a drug-free Baseline session; one week later at a Treatment session, at which the EEG protocol was administered 2.5 h after participants had taken an inactive placebo or 10 µg LSD base sublingually; and six weeks later at a drug-free Final session, two days following their last of 14 doses. Interventions were identical aside from the contents of the dose. Participants were instructed not to drink alcohol for 24 h before the EEG recording and to drink their usual amount of caffeine that morning. EEG recordings took place either in mid-morning or early afternoon and were conducted for each participant at the same time of day wherever possible.

### EEG acquisition and pre-processing

In brief, 64-channel EEG recordings were collected using Brain Vision Recorder, and Brain Products actiCAP electrodes with Brain Products MRPlus amplifiers. See Additional file 1 for detailed specifications of EEG acquisition and pre-processing.

### LTP protocol

**Fig. 1:**
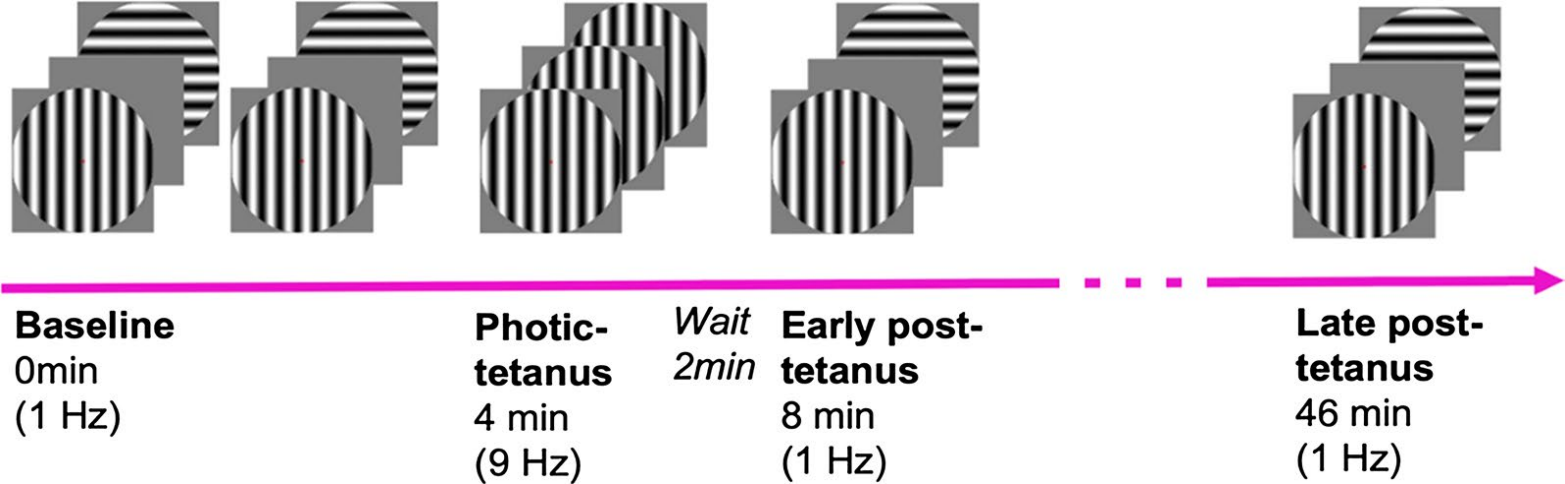
Diagram of the LTP paradigm timing and sequence. Adapted from Sumner, McMillan et al. (2020)

Participants were presented with intermittent horizontal and vertical circular sine contrast gratings in four phases, (Fig. 1) with a red fixation dot in the centre of the screen throughout each phase. The stimulus subtends 8 degrees of visual angle when participants are positioned 90 cm in front of the screen, with distance confirmed prior to each phase. In the baseline phase, participants are shown horizontal and vertical sine gratings in a random order 120 times each at low frequency of 1 Hz. Stimuli are on the screen for a duration of 34.8 ms, with interstimulus intervals randomised between five intervals from 897 to 1036ms which occurred equally often, for a total duration of 4 min [21]. This phase is followed by the photic-tetanus phase, in which one orientation is tetanised at a high frequency (9 Hz) 1000 times for a duration of 2 min to induce LTP. Orientation of tetanised stimuli is counterbalanced between participants. Following a two-minute delay, the baseline 1 Hz frequency presentation is repeated as the early post-tetanus phase, and again 40 min later in the late post-tetanus phase. To record STP and e-LTP respectively.

### LTP analysis

Separate analyses were planned for the early and late contrasts of Baseline vs. Treatment (acute) and Baseline vs. Final (sustained) visit. Participants were excluded from each analysis if data at either point was missing or corrupted (three participants in the Baseline vs. Treatment and none in Baseline vs. Final). In the Baseline vs. Treatment analysis, they were also excluded if the interval between Baseline and Treatment was less than one week (four participants), due to increased risk that insufficient time for the effects of the first potentiation of the task to have dissipated. In the Baseline vs. Final analysis, they were excluded if the Final recording was not two days after their last dose (21 participants), or if they had more or less than 14 doses total (eight participants). See Murphy et al.  [4] for details regarding late EEG sessions due to Covid-19-related disruptions.

Difference waves were computed for the Early (2 min post-tetanus minus pre-tetanus) and Late (40 min post-tetanus minus pre-tetanus) timepoints using FieldTrip 2016 [44] and subsequent analysis done in SPM12 [45, 46]. The advantage of SPM12 is that it represents spatio-temporal data as a continuous statistical parametric map and then identifies clusters of significance in both time and space, using random field theory to perform familywise error correction (FWE-c). This allows for large regions of interest (ROI’s) and objective identification of time windows, while still controlling for multiple comparisons.

Based on previous LTP research [21, 22], an occipital-parietal ROI was defined, consisting of electrodes P1, P2, P3, P4, P5, P6, P7, P8, Pz, PO3, PO4, PO7, PO8, PO9, PO10, POz, O1, O2 and Oz. Initial parameter-finding analyses of Time (Early/Late) x Stimulus (Tetanised/Non-tetanised) were conducted in SPM12 on each analysis with planned contrasts to confirm LTP was occurring (Additional file 1: Table S1), identify the time windows of interest, and test for specificity (altered potentiation of the Tetanised vs. Non-tetanised stimulus). No contrast was made by Group or Session. Based on the existing literature, contrasts were refined to specifically look for lateralised negative components in the early condition (N1/b) and central positive components (P2) in the late condition [14]. Planned contrasts were one-tailed *t*-tests and effects were considered significant if the FWE-c *p*-value was < 0.05. As specified in the introduction we hypothesize that there will be increases in P2 modulation at the measure session and that there may also be an increase in N1b amplitude. We will analyse the treatment session the same way, though this was exploratory and we had no specific hypotheses.

Time windows were selected by extracting the cluster windows around significant peaks from the parameter-finding analyses (negative lateralised peaks in the Early condition and positive central peaks in the Late condition). These were then used in the main analyses of a repeated measures ANOVA of Group (Placebo vs. LSD) x Session (Baseline vs. Treatment/Final) in separate Early and Late analyses. Main and post-hoc effects were considered significant if they achieved a FWE-c *p*-value < 0.05, while interaction effects were less conservatively considered significant with an uncorrected *p*-value < 0.001, in line with previous literature [22]. In both cases FWE-c *p-*values are reported, and these values are not further corrected in the post-hoc tests. Where a cluster presents multiple peaks, the most significant peak is reported.

In summary, the main statistical plan consisted of four separate 2 × 2 ANOVA as described in Table 1.

**Table 1:** Factors, difference waves, and components of interest for main analyses

	Factor 1: Group	Factor 2: Session	Difference wave	Component of interest
1.	Placebo vs. LSD	Baseline vs. Treatment	Early (Post1-Pre)	N1b
2.	Placebo vs. LSD	Baseline vs. Treatment	Late (Post2-Pre)	P2
3.	Placebo vs. LSD	Baseline vs. Final	Early (Post1-Pre)	N1b
4.	Placebo vs. LSD	Baseline vs. Final	Late (Post2-Pre)	P2

### Computational modelling

**Fig. 2:**
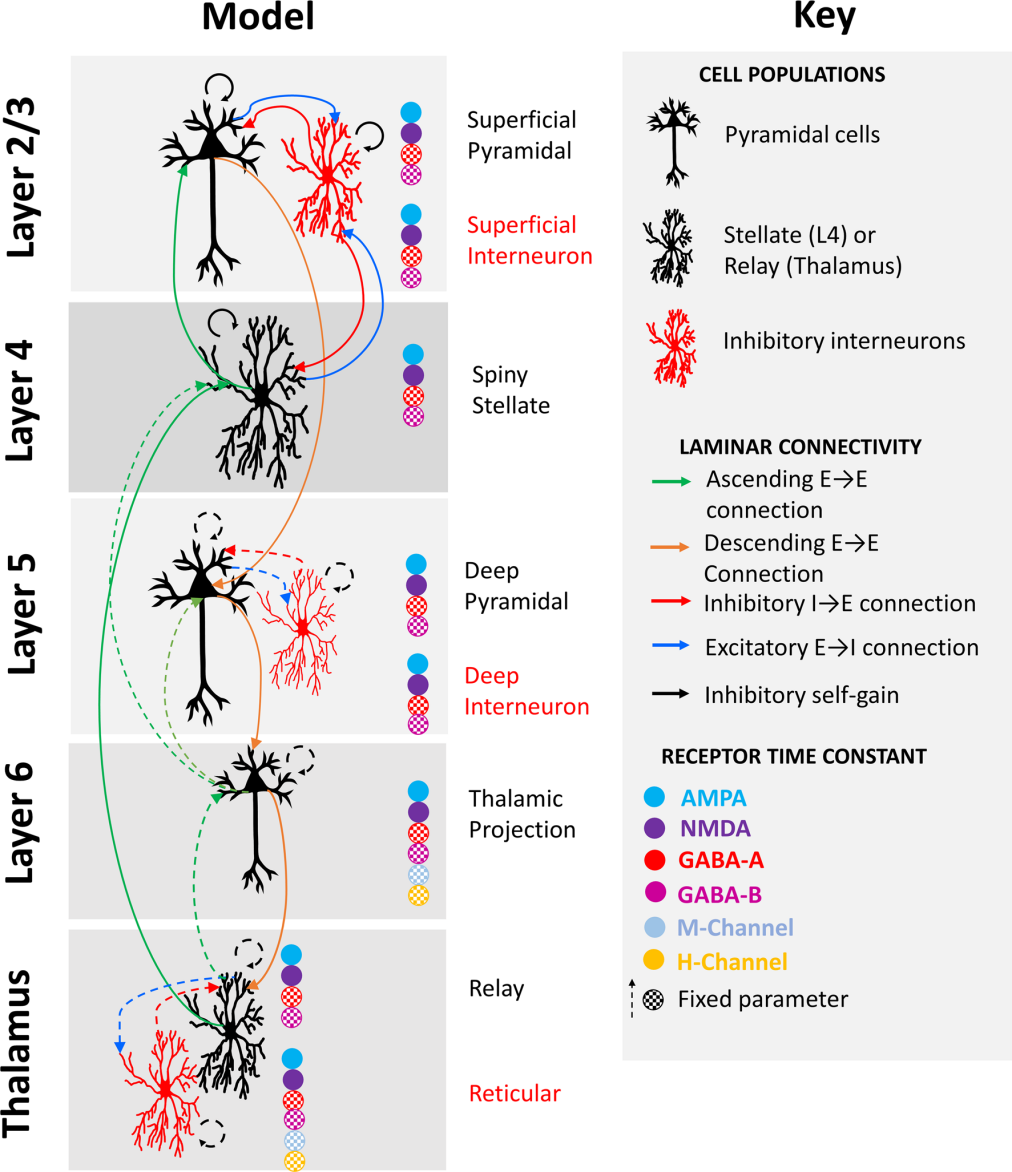
TCM architecture and connectivity with six cortical column neural populations and two thalamic populations. Cortical populations include layer 2/3 superficial pyramidal (SP) and superficial interneuron (SI) cells, layer 4 spiny stellate cells (SS), layer 5 deep pyramidal (DP) and deep interneuron (DI) cells, and layer 6 thalamic projection (TP) cells. Thalamic populations include reticular (RT) and relay cells (RL). Connectivity between cells include ascending (in green) and descending (in orange) connections between excitatory cell populations, and inhibitory (in red) and excitatory (in blue) connections between excitatory and inhibitory cell populations. Solid lines indicate connectivity parameters which were allowed to change within the model, and dashed lines indicate fixed parameters. The model also parameterises the decay constants of AMPA, NMDA, GABA-A, GABA-B, M- and H-channels

In this study we implemented a TCM as in Stone et al. [22]. and Sumner et al. [36]. Source analysis of the ERP data was used to identify the peak of effects and isolate a 5 mm spherical radius around this voxel which was then isolated from each individual participant’s data as a virtual local field potential (LFP; full description in the Additional file 1). The TCM was fitted to the EEG data extracted into the virtual LFP using DCM (implemented as standard in SPM12). The data were fitted to Baseline and Treatment datasets (73 participants) and Baseline and Final datasets (51 participants) separately to look at acute and sustained drug effects independently. Figure 2 depicts the model architecture, with parameters which were allowed to vary (to describe task and drug related effects) given as solid lines, and those which are fixed given as broken lines. The model parameterises the interlaminar connections between: superficial pyramidal (SP) cells, superficial interneurons (SI), spiny stellate (SS), deep pyramidal (DP) cells, deep interneurons (DI), thalamic projection (TP), and thalamic reticular (RT) cells and relay (RL) cells. The model also parameterises the decay constant of AMPA, NMDA, GABA-A, GABA-B, and M- and H- channels.

A parameterised general linear model was incorporated into the inversion protocol. A linear change from pre-tetanus that is greatest in the late post-tetanus block was entered to reflect e-LTP. Linear contrast: [− 1 0 1]. A non-linear change from baseline that peaks in the first post-tetanus block (to model general excitability and STP) was also entered. Non-linear contrast: [− 1 1 0]. This was combined in the contrast [− 1 1 0; −1 0 1] allowing for both non-linear and linear contributions to describe the condition-specific effects and representing the model that best fit to describe visually induced LTP.

Analysis of the parameter differences was conducted using parametric empirical Bayes (PEB) [47] in a ‘PEB of PEBs’ method [48] which is able to account for both the within-subjects contrasts of Session, and the between-subjects contrasts of Group (full description in Additional file 1).

## Results

### Parameter-finding

Parameter-finding analyses of the time windows for analysis are given in the Supplemental Materials. Time windows of 160–200 ms in the Early condition including tetanised stimuli only, and 170–250ms in the Late condition with both tetanised and non-tetanised stimuli averaged were chosen based on these analyses.

### Acute effects

The dataset for the Baseline vs. Treatment analysis was 73 out of 80 participants – placebo *n =* 36 and LSD *n* = 37.

#### Early potentiation

In the Baseline vs. Treatment analysis of the Early condition difference waves, an interaction effect of Group x Session was found in a right-lateralised cluster peaking at 198ms (*F*_(1,142)_ = 13.05, *p =* 0.0201 FWE-c). No main effect of Session was found, but a lateralized main effect of Group was found peaking at 175 ms in the left hemisphere (*F*_(1,142)_ = 18.48, *p =* 0.0022 FWE-c) and 170 ms in the right hemisphere (*F*_(1,142)_ = 15.80, *p =* 0.0066 FWE-c) in a distinct cluster from the interaction effect. Post-hoc tests showed that this main effect was driven by less potentiation in the LSD group relative to placebo with matching peaks in the left hemisphere at 175ms (*t*_(142)_ = 4.30, *p* = 0.0011 FWE-c) and in the right hemisphere at 170ms (*t*_(142)_ = 3.97, *p* = 0.0033 FWE-c). This indicates that potentiation in the LSD group was lower than in the placebo group, irrespective of whether the drug had been given or not.

**Fig. 3:**
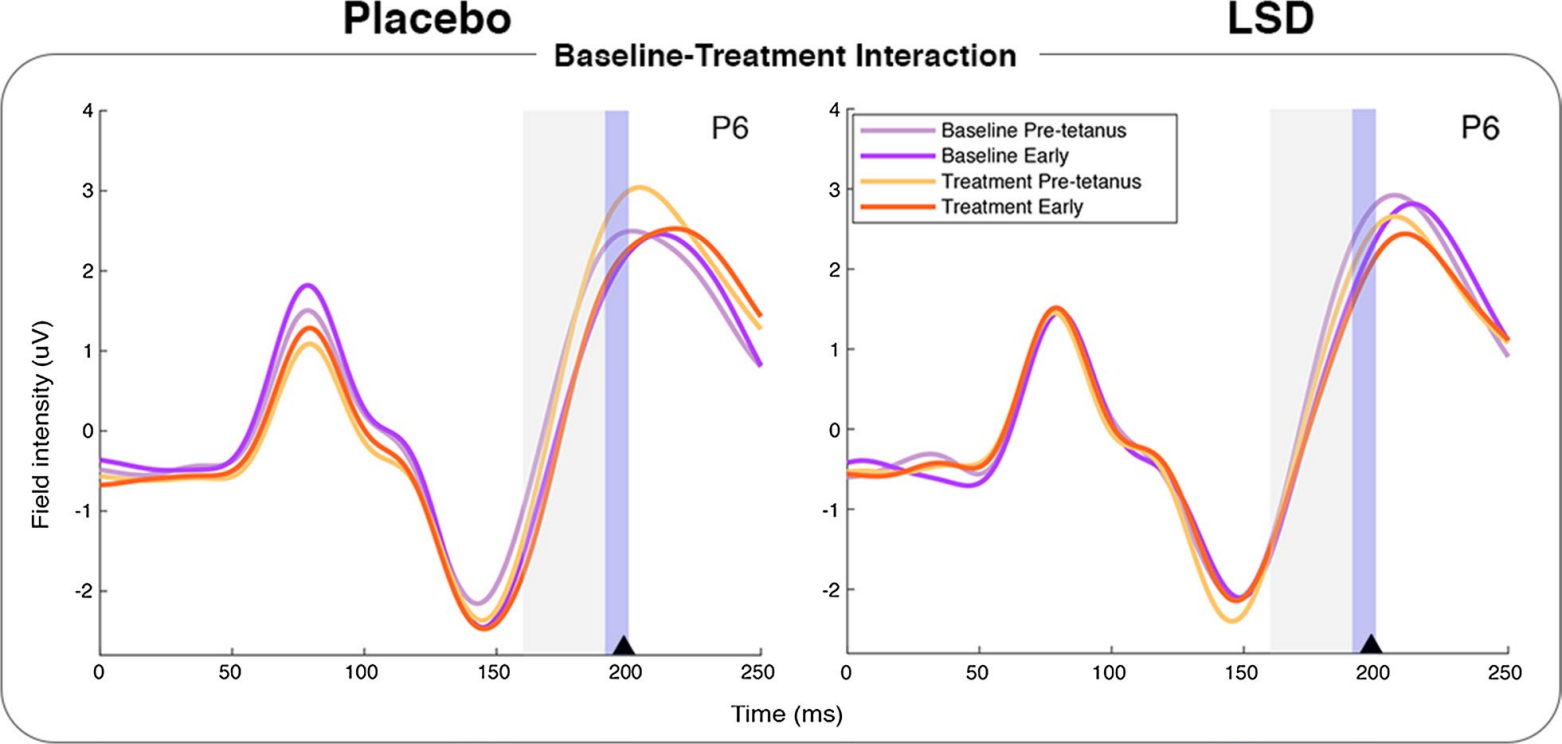
Interaction of Group x Session in the Early Baseline to Treatment analysis as illustrated at electrode P6. ERPs shown are pre-tetanus and early post-tetanus, arrow shows peak of interaction significance, blue shaded area shows time window cluster around peak, grey shaded area shows the analysis parameter window. P6 here is illustrative only, analysis was conducted on a 19 electrode occipital-parietal ROI.

Post-hoc tests of the interaction effect showed no significant within-group difference in the LSD group in the region of interest, however there was a significant difference within the placebo group, with a more negative component in the Treatment session than the Baseline, peaking in the right hemisphere at 200ms (*t*_(142)_ = 3.92, *p* = 0.0039 FWE-c). This effect had an identical maximum intensity projection (MIP) to the original interaction (Additional file 1: Fig. S3). There were no significant between-groups differences in this region. Inspection of the raw ERP at this time point (Fig. 3) shows that rather than falling within the trailing edge of the N1 (typically the N1b is defined as being between the peak of the N1 and halfway to the peak of the P2 [49]), the interaction cluster falls just prior to the peak of P2.

#### Late potentiation

In the Baseline vs. Treatment analysis of the Late condition difference waves, no interaction of Group x Session was found, nor any main effect of Group, however there was a main effect of Session, with a central cluster peaking at 192ms (*F*_(1,142)_ = 15.6, *p* = 0.0153 few-c), consistent with the P2 component. Post-hoc tests showed that the amplitude of the overall difference wave was greater in the Baseline condition over the Treatment condition (*t*_(142)_ = 3.95, *p* = 0.0077 FWE-c). This indicates that the increase in P2 amplitude post-tetanisation was lower on the Treatment day than the Baseline day, regardless of Group.

### Sustained effects

The sample size for the Baseline vs. Final analysis was 51 out of 80 – placebo *n* = 26, LSD *n* = 25.

#### Early potentiation

In the Baseline vs. Final analysis of the Early condition difference waves, no interaction effect of Group x Session was found, nor main effects of Group or Session. This indicates that N1b potentiation was no different at the Final visit than it was at Baseline for either group.

#### Late potentiation

**Fig. 4:**
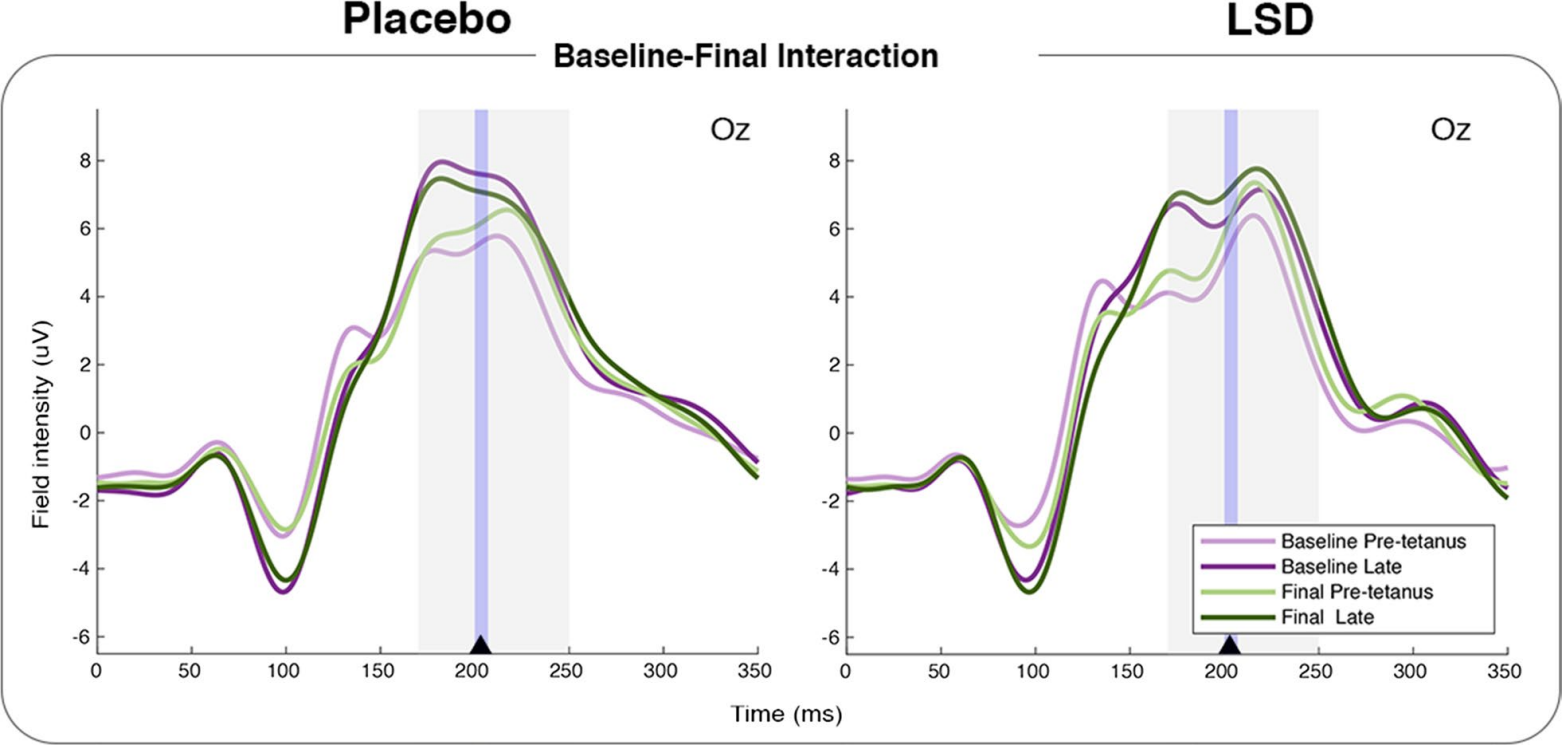
Interaction of Group x Session in the Late Baseline to Final analysis as illustrated at electrode Oz. ERPs shown are pre-tetanus and late post-tetanus, arrow shows peak of interaction significance, blue shaded area shows time window cluster around peak, grey shaded area shows the analysis parameter window. Oz here is illustrative only, analysis was conducted on a 19 electrode occipital-parietal ROI.

In the Baseline vs. Final analysis of the Late condition difference waves, an interaction effect of Group x Session was found with a lateralised peak in the left hemisphere at 203 ms (*F*_(1,98)_ = 12.16, *p* = 0.0596 FWE-c and *p* = 0.0007 uncorrected). Inspection of the topographies show that while lateralised, this difference did relate to the lateral edges of a positive centralised component consistent with the P2. No main effect of Group was found, but there was a significant main effect of Session with a peak at 222 ms (*F*_(1,98)_ = 34.07, *p* < 0.0001 FWE-c). Post-hoc tests showed no effect of Group at Baseline or the Final visit, and no effect of Session in the LSD group, but a significant effect of Session in the Placebo group with a peak at 228 ms (*F*_(1,98)_ = 29.27, *p* = 0.0001 FWE-c). Further one-tailed *t*-tests showed that this was driven by the placebo group’s difference wave being significantly more positive at the Baseline visit than at the Final visit (*t*_(98)_ = 5.41, *p* = 0.0001 FWE-c) (Fig. 4). This indicates that while there was no difference in the late potentiation of the P2 between groups at Baseline, and no within group difference in the LSD condition, there was a significant within group difference for the placebo group. This was driven by less potentiation of the P2 in the placebo group at the Final visit than there was at Baseline.

### Computational modelling

**Fig. 5:**
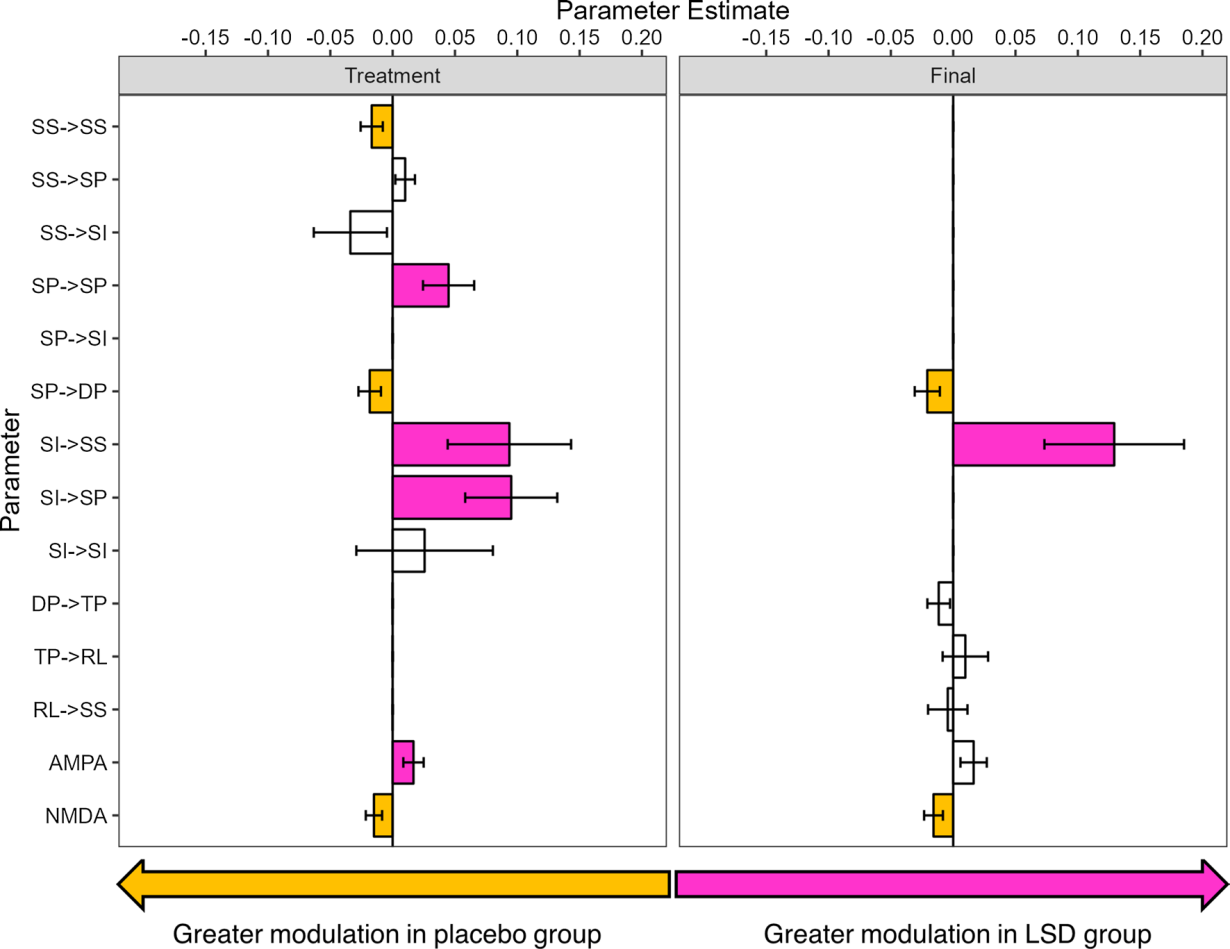
Parameter estimates of difference in LSD group relative to placebo in the Baseline to Treatment and Baseline to Final visits. Coloured bars indicate very strong evidence (> 0.99 poster probability) with pink (positive values) indicating a greater visual LTP mediated change in that parameter for the LSD group over placebo and yellow bars (negative values) indicating greater visual LTP mediated change in that parameter in placebo over LSD. Error bars indicate standard deviation

**Fig. 6:**
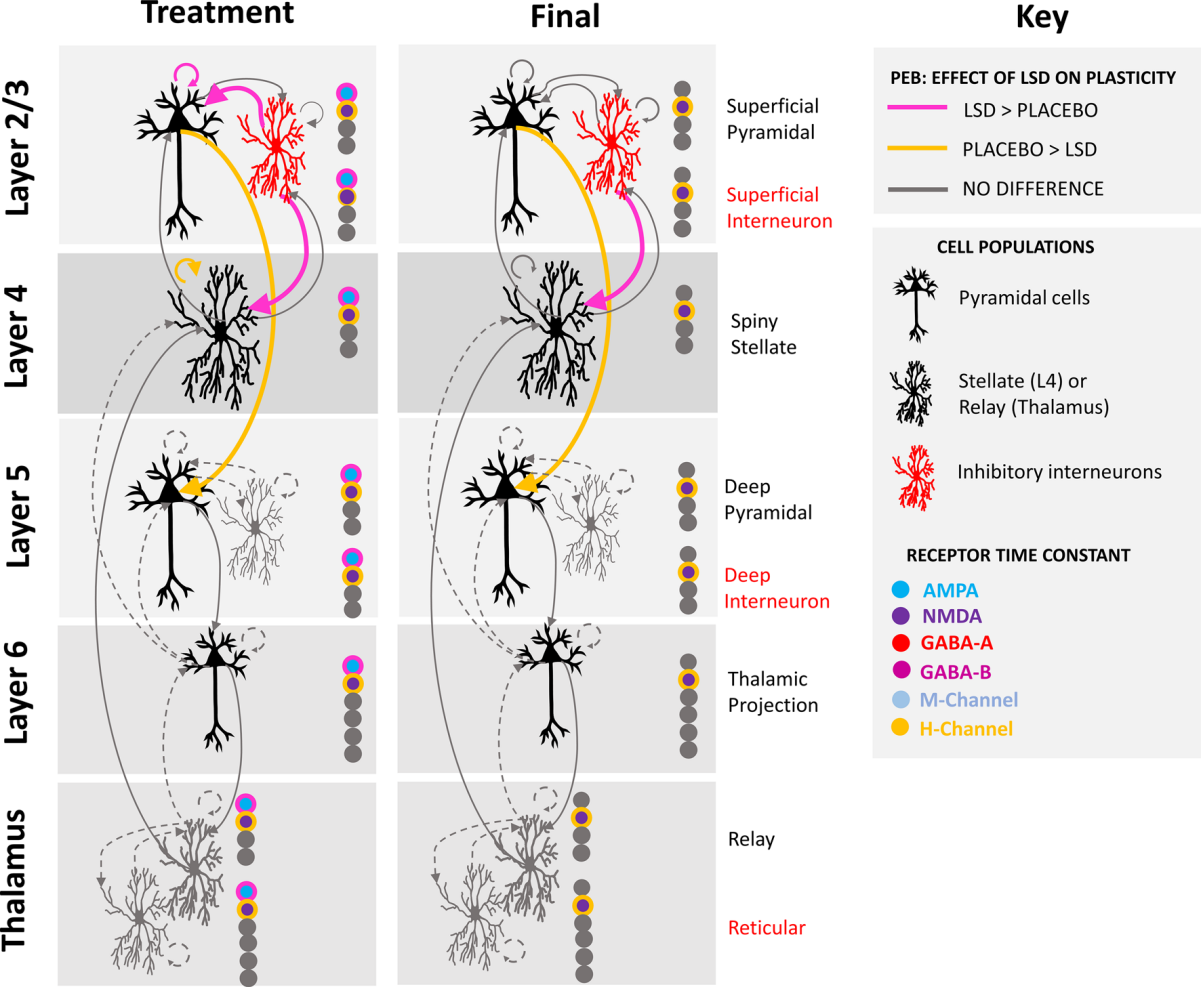
Parameters with very strong evidence of the difference between LSD and placebo groups in the PEB of PEBs for the Baseline vs. Treatment and Baseline vs. Final analyses

The final re-estimated DCM provided an excellent fit for the Baseline vs. Treatment and Baseline vs. Final analyses (> 99% variance explained). Figure 5 shows the parameter estimates with the parameters which survived the threshold for ‘very strong’ (posterior probability > 0.99) evidence of differences in the LSD group relative to placebo. Figure 6 shows these differences in context of the model and Additional file 1: Table S7 gives them in tabular form.

In the Baseline vs. Treatment analysis, the parameters which showed a greater modulation in the LSD group relative to placebo were inhibitory SI→SP, SI→SS, and intrinsic SP→SP connections, as well as AMPA channels. Those which showed greater modulation in the placebo group relative to LSD were excitatory feedforward SP→DP, and inhibitory intrinsic SS→SS connections as well as NMDA channels. In the Baseline vs. Final analysis, the SI→SS change remained greater in the LSD group and the SP→DP and NMDA changes remained higher in the placebo group.

## Discussion

This study analysed the change in ERP components associated with LTP at a drug-free Baseline session, at a Treatment session 2.5 h after administration of an LSD microdose or inactive placebo, and at a drug-free Final visit following 6 weeks of microdosing every three days. Comparison of the Baseline and Treatment sessions showed no difference within the LSD group. The placebo group showed greater potentiation at Treatment compared to Baseline, however the affected ERP component is unclear. While in the same area of the N1b (most proximal to electrode P6) it in fact falls out of the N1b time window and is a change in positivity between components. Comparison of the Baseline and Final sessions in the Late condition showed a difference in P2 potentiation within the placebo group, with potentiation in the placebo group being lower at the Final session than it was at Baseline. Changes in the placebo group were unexpected, made more difficult to interpret given no specific ERP component changed in the Baseline to Treatment analysis.

We source localised a single LFP in the visual cortex and fit a thalamocortical model to each condition over the entire post-stimulus time window (0-350 ms). Given there was no manipulation or intervention in the placebo group, differences between placebo and LSD over sessions can be related to physiological parameter differences related to drug/no-drug. Thus, overcoming the difficulty with interpreting the electrode-level SPM analysis through ERP modulation differences. Differences were found between groups. Post-treatment, the LSD group had greater modulation of inhibitory input into layer 4 spiny stellates, superficial pyramidal cells and greater modulation of superficial pyramidal self-gain. Contrastingly the placebo group had relatively greater modulation of the self-gain of spiny stellates and superficial pyramidal input into layer 5 deep pyramidal cells. While AMPAR decay constant modulation was greater in the LSD group, the placebo group had greater NMDAR decay constant modulation.

By the Final session, modulation of superficial pyramidal input into layer 5 deep pyramidal cells, and the NMDAR decay constant remained relatively greater in the placebo group. Inhibitory input into spiny stellates remained greater in the LSD group. Differences in modulation of self-gain in the superficial pyramidal, and spiny stellate cells was no longer seen, nor were the inhibitory inputs to the superficial pyramidal cells and the AMPAR decay.

### Evoked response outcomes

This study did not reproduce the increased potentiation of the P2 component previously seen following a full dose of the psychoplastogen ketamine [21], for which there are several possible explanations. Notable is that the EEG recordings in the ketamine study were conducted in the early post-acute phase of the drug, whereas in the present study they were timed to coincide with the peak of subjective effects, and in a late post-acute phase two days later. Previous studies have demonstrated that while an upregulation on pro-plastic genetic factors can be seen in the acute phase of psychoplastogen administration, enhancements of circulating BDNF in humans [50] and structural plasticity in animals [51] have only been demonstrated post acutely (4+ and 6+ h, respectively), suggesting that the Treatment recording in our study may have been too early (2.5 h post-dose) to capture these effects. However, the Final recording fell two days after the final dose, and also did not capture this effect (while there was an interaction, it was driven by changes in the placebo group). Finally, this previous finding was in a clinical population with MDD [21]. Pre-clinical evidence has demonstrated differential responses of stressed vs. non-stressed mice, suggesting that behavioural effects of altered neuroplasticity may not be observable in healthy populations [52]. Finally, that the doses used in the present study were very low, while those used in the ketamine study were full doses. In order to understand this relationship, comparably high LSD doses would need to be tested with the LTP paradigm.

### Evidence for microcircuitry changes

LTP-induced modulation of feedforward connections from the superficial (layer 2/3) to deep (layer 5) pyramidal cells was seen to be lower in the LSD group than the placebo group. Within a model of predictive coding, these connections are thought to convey feedforward prediction error from lower sensory-processing areas of the cortex to higher areas in which predictive models are generated (and from which expectations are then propagated back down) [32]. One of the proposed mechanisms of the high-level effects of psychedelics on the brain is via the disruption of these hierarchical predictive coding models, such that top-down (feedback) predictions are relaxed in favour of sensitivity to bottom-up (feedforward) information [53]. In a drug-free administration of the LTP paradigm [36], this parameter was significantly decreased following tetanisation, so reduced modulation by LSD may indicate maintenance of a sensitivity to feedforward connections.

An increased modulation of connections that regulate the excitatory activity of superficial pyramidal cells was seen under LSD in the acute, but not sustained, analysis with modulation of inhibitory inputs from superficial interneurons, as well as intrinsic self-gain connections being greater under LSD than placebo. Of these, superficial pyramidal self-gain has been shown by previous application of the same TCM model to contribute to LTP [36]. Alteration of the activity of these cells may be driving the altered connectivity of the feedforward connections to layer 5 mentioned above [32]. Both connections are parameterised as GABAeric inhibition; LSD is known to acutely suppress neuron firing and increase serotonin (5-HT) levels. This leads to an increase in GABAergic interneuronal activity via 5-HT_2A_ and 5-HT_2C_ receptors [54]. Suppression of 5-HT firing by LSD occurs even at low doses [42].

Interneuronal input into spiny stellates in layer 4 is a feedback parameter that has been shown to be increased by LTP [36] and was modulated more strongly by LSD than placebo in the acute session and the final session relative to baseline. Placebo was associated with greater modulation of self-gain on stellates in later 4. Modulation of inhibitory interneuronal input into stellates was also seen in a previous application to this model to LTP sessions conducted over the preserved menstrual cycle of women on hormonal birth control, with a reduction seen in the perimenstrual-like relative to the mid-follicular-like phase [22]. GABAergic interneuronal inhibition has been shown to provide a gating mechanism preventing the induction of LTP from layer 4 to 3 [55]. In that study, a reduction in inhibitory gating was accompanied by increased P2 potentiation in the perimenstrual phase. In the current study, in the Final session analysis there was significantly reduced P2 potentiation in the placebo but not LSD group. Greater AMPAR and lower NMDAR decay modulation in the LSD group may be consistent with pre-clinical findings that administration of repeated low doses of LSD to mice causes potentiation of AMPAR but not NMDAR synaptic responses in medial prefrontal cortex pyramidal neurons [52].

Overall the results from the modelling are consistent with LSD modulating neural plasticity. Invasive and previous modelling studies support the interpretation that changes in modulation of GABAergic connections may be related to LSD driven decreases in neuronal firing that increase GABAergic inhibition. Further supported by the finding these parameters are not modulated in the final session, two days after the last dose. By contrast modulation of interneuronal input into layer 4 and superficial pyramidal input into layer 5 deep pyramidal neurons are consistent with the REBUS model and LSD driving greater sensitivity to feedforward connections. Incorporating both feedforward and intrinsic connections has been shown to be the model of best fit for Hebbian learning via LTP when compared to predictive coding (which favours feedforward and feedback connections). In sum this suggests that LTP triggers alterations in feedforward Hebbian learning (Spriggs, Sumner et al. 2018, Sumner, Spriggs et al. 2018) and that LSD supports this process.

However, despite the plausibility of the modelling results, it is interesting that so many effects on the laminar connectivity were contained in the time course of the primary cortex LFP modelled, but did not produce a clearly corresponding result in the ERPs. This may speak to the strength and higher sensitivity of the modelling approach, however it also invites caution with interpreting these results unless they reliability and generalisability is demonstrated in future studies.

### Strengths, limitations and future directions

Identified in the discussion, we may have timed the visual LTP task too early to capture changes to LTP-mediated plasticity. The dose may also be too small to produce detectable shifts in plasticity. To the authors’ knowledge, no study has successfully implemented the visual LTP paradigm in a serotonergic psychedelic study using macro or medium doses. Studies in the dose/visual LTP sensitivity relationship are needed, as are studies varying the post-drug time-window tested. These could be informed by animal studies into LTP modulation and the time-course of psychedelic effects on plasticity.

A strength of this research is that, in contrast subjective ratings of participant experience, the visual LTP paradigm is objective and unlikely to be subject to expectancy effects. Expectancy and unblinding have been identified in prospective studies as high within the populations who microdose, and able to affect participants’ subjective experiences [56–58]. Expectancy and unblinding in regards to the current trial have been discussed in previous publication of data from this cohort [4].

While significant interaction effects were found, unexpectedly the amplitude of the difference waves of the placebo group varied significantly between sessions in the acute early phase (Treatment potentiation increased in a positivity after the N1b), and the follow-up late phase (Final P2 potentiation reduced), while the LSD group did not. Given the study was a randomised trial there was no systematic difference for placebo participants that can explain this. However, it does raise the question of the test-retest reliability of the Teyler protocol, for which three repeated measures has rarely been employed previously (one example: [22]). Outside of this study counterbalancing has always been applied in our own groups’ research to mitigate order effects as a potential confound. In general, VEP amplitudes have low intra-individual variability over time [59, 60], however the reliability of induced modulation of the VEP has not been tested.

There also remains the question of why the placebo group variation was significantly different from the LSD group. It’s possible the variation is not very strong and is a type 1 error in the placebo group. This is supported by the lack of clarity as to which component is more potentiated in the Treatment session (it does not map neatly only N1b or P2). Else if there was some unknown variable that we assume affected both groups equally then it could be interpreted, cautiously, as a drug-related effect. For example, it could be that LSD maintained/promoted stability in VEP post-tetanus modulation across the Baseline to Treatment and Baseline to Final visits. A potential mechanism of this could be habituation to the induction of LTP [61]. However, there is insufficient evidence to speculate further, including on any mechanism that may drive this. While previous research has not seen such an effect, previous studies have always counterbalanced repeated sessions. It will be interesting to see if the effect is replicated in other work. Further studies ought to investigate the stability of visually induced LTP in repeated measures protocols. Particularly determining whether there is an ideal time to leave between session.

Modelling EEG data from the visual LTP task using a virtual LFP extracted from source localised data has strengths. Conceptually the parameters assessed are most like how invasive animal studies implant and record LTP which makes it easier to relate results to the known pharmacodynamic effects of drugs, and electrophysiological phenomena such as LTP. Further, it assesses the entire time course of the evoked response rather than discrete VEP components. However, a model is always limited to assess only the parameters it computes. In particular, in the study of psychedelics, a limitation of the application of the TCM to this data is that it does not incorporate a 5-HT2A receptor parameter. This receptor has a well-established role as the driver of subjective and behavioural effects of serotonergic psychedelics, and its inclusion could be used to optimise the model to capture the effects of these drugs [62].

Additionally, the DCM was conducted on the source-localised peak of LTP effects in the visual cortex, and as such it would be useful to test whether the interlaminar connectivity changes observed are generalisable to other areas of the brain. Further corroboration with other non-invasive LTP models such as tactile, auditory, and TMS stimulation under microdoses of LSD could be useful in establishing the generalisability of this effect, as well as correlation of LTP effects with higher-order neuroplastic functions such as memory performance, as has been done under drug-free conditions [23].

## Conclusions

While analysis of ERP peaks did not show any effect of LSD on LTP, modelling of the entire waveform was able to detect functional changes in connectivity within the cortical column on the visual cortex. These changes suggest LSD may play a role in altering feedforward neural plasticity during the LTP task. This modelling approach may be more appropriate for examination of the effects of low doses of psychedelics than traditional ERP peak analyses, give its increased sensitivity.

### Supplementary information

**Additional file 1. **Additional Methods (Sample Size; Exclusion Criteria; EEG Pre-Processing; Parameter-Finding Planned Contrasts; Source Analysis; Thalamo-cortical Model; Parametric Empirical Bayes) and Additional Results (Demographics; Parameter-Finding Results; VEP Components)

## Data Availability

The datasets used and/or analysed during the current study are available from the corresponding author on reasonable request. Code for the TCM model is available here: https://github.com/alexandershaw4/LTP_code.

## Abbreviations

5-HT: Serotonin
DCM: Dynamic causal modelling
DI: Deep interneuron cell
DP: Deep pyramidal cell
EEG: Electroencephalography
ERP: Event-related potential
FWE-c: Family-wise error corrected
LFP: Local field potential
LSD: Lysergic acid diethylamide
LTP: Long-term potentiation
MDD: Major depressive disorder
MIP: Maximum intensity projection
PEB: Parametric empirical Bayes
RL: Relay cell
RT: Reticular cell
ROI: Region of interest
TP: Thalamic projection cell
SI: Superficial interneuron
SP: Superficial pyramidal cell
SS: Spiny stellate cell
TCM: Thalamo-cortical model
VEP: Visual evoked potential
