# Supplementary material for: Modulation of long-term potentiation following microdoses of LSD captured by thalamo-cortical modelling in a randomised, controlled trial
Source: BMC Neurosci. 2024 Feb 5;25:7. doi: 10.1186/s12868-024-00844-5 (PMC10845757; doi:10.1186/s12868-024-00844-5)
Supplement: Supplementary file 1 — Additional file 1. Additional Methods (Sample Size; Exclusion Criteria; EEG Pre-Processing; Parameter-Finding Planned Contrasts; Source Analysis; Thalamo-cortical Model; Parametric Empirical Bayes) and Additional Results (Demographics; Parameter-Finding Results; VEP Components) [file 12868_2024_844_MOESM1_ESM.pdf]

Additional file 1: Modulation of long-term potentiation following microdoses of LSD captured by thalamo-cortical modelling in a randomised, controlled trial.

Robin J. Murphy[1*], Kate Godfrey[2], Alexander D. Shaw[3], Suresh Muthukumaraswamy[1], and Rachael L. Sumner[1]

[1]School of Pharmacy, University of Auckland, Auckland, New Zealand

[2]Centre for Psychedelic Research, Department of Psychiatry, Imperial College London, London, UK

[3]Department of Psychology, Exeter University, Exeter, UK

* Corresponding author: Robin J. Murphy - robin.murphy@auckland.ac.nz

Table of Contents

Additional Methods 3

Sample Size 3

Exclusion Criteria 3

EEG Pre-Processing 5

Parameter-Finding Planned Contrasts 6

Source Analysis 7

Thalamo-cortical Model 7

Parametric Empirical Bayes 7

Additional Results 9

Demographics 9

Parameter-Finding Results 10

VEP Components 11

References 14

Additional Methods

Sample Size

Sample size calculations were described in a pre-registered protocol paper (1). Recruitment was halted when the sample size of 80 (LSD = 40, placebo = 40) was complete. Following screening, participants were allocated into placebo or LSD groups in a 1:1 ratio in blocks of 10 via a computer-generated random sequence by an independent scientist who had no contact with participants and no further involvement in the trial. All study team members and participants were blinded. The first three blocks of 10 were unblinded to the study team following completion of all participants in the block. The following five blocks remained blinded to the study team until the completion of the entire trial. All participants remained blinded to their condition until the completion of the entire trial. Further description of randomisation and blinding is given in the protocol paper and in the first published paper of results (1, 2).

Exclusion Criteria

Table S1: Full inclusion criteria

	Inclusion criteria
Consent	Willing and able to give informed consent for participation in the trial, reconfirmed verbally at each study visit.
Demographics	
Age	25-60 years
Sex	Male

Table S2: Full exclusion criteria

	Exclusion criteria
Consent/communication	Inability to speak or read English
Physiological health	
Diagnosis	Unstable medical or neurologic condition as assessed by study physician
Lab work	Significant renal or hepatic impairment
Vital signs	Cardiovascular conditions including abnormal heart rate seen by ECG

	Resting blood pressure not exceeding 160 mmHg systolic and 90 mmHg diastolic<br>Body weight between 50-120 kg
Medical history	Contraindications for MRI scanning
Mental health	
Diagnosis	Lifetime history of major depressive disorder, schizophrenia, or other psychotic disorders, or bipolar I or II disorder as assessed by the Mini International Neuropsychiatric Interview (MINI)<br>Current diagnosis of PTSD, anxiety and panic disorders, OCD, dysthymic disorder, anorexia, and bulimia as assessed by the Standard MINI
Current risk	Elevated of suicide as determined by study psychiatrist using the Columbia-Suicide Severity Rating Scale (C-SSRS)<br>Elevated risk of developing psychosis as determined by study psychiatrist using the Comprehensive Assessment of At Risk Mental States (CAARMS)
Family diagnosis	First degree relatives diagnosed with schizophrenia or other primary psychotic disorder, or bipolar I or II disorder
Medication	Current use of any prescribed psychotropic medication
Substance use	Substance use disorder in the previous 3 months as assessed with a New Zealand modified version of the NM-ASSIST<br>Failed breathalyser and/or multipanel drug urine tests at screening with one follow up in trial<br>Use of serotonergic psychedelic drugs in the last year<br>Lifetime history of psychedelic microdosing

No strict exclusion for tobacco or alcohol use was included (aside from substance use disorder indicated by the NM-ASSIST), however excessive use of either was considered holistically by clinicians when appraising the participant’s overall health.

Study Site

EEG data was collected at the Clinical Research Centre at the University of Auckland in specialised clinic rooms.

EEG Acquisition

EEG acquisition and pre-processing replicated the methods previously reported in our lab (3). EEG acquisition hardware and software are outlined in Table S3. Simulus events and EEG recordings were synchronised through TTL pulses via the parallel port of the display computer. Participants were situated 90cm in front of the display monitor, with distance checked between recordings. Electrode impedance below 10 kΩ was achieved prior to recording. Electrode FCz was used for the online reference, and AFz for the ground.

Table S3: EEG acquisition hardware and software

Function	Product
Hardware	
Caps	actiCAP Ag/AgCl active shielded electrodes
Amplifiers	Brain Products MRPlus
Display	ASUS VG248QE computer monitor –1920 x 1080 resolution, 144 Hz refresh rate
Software	
EEG recording	Brain Vision Recorder (Brain Products GmbH, Germany) – 1000Hz sample rate, 0.1µV resolution.
Stimulus display	MATLAB (The Mathworks Inc., Natick, MA) and the Psychophysics Toolbox

EEG Pre-Processing

EEG pre-processing used the Fieldtrip toolbox. Firstly, data was epoched into time windows of -200ms pre- and 500ms post-stimulus onset, with the 200ms prior to stimulus onset used for baselining. Ocular artifacts were removed by first using a semi-automated process, followed by manual artifact rejection. A modified version of the Fieldtrip semi-automated electro-occulogram (EOG) artifact rejection tool (ft_artifact_zvalue) was applied to frontal electrodes Fp1 and Fp2. Additionally, a second order 0.3-1Hz Butterworth bandpass filter was applied with 150ms of negative trial-padding to eliminate only trials in which blinks occurred during the stimulus display. Manual artifact rejection was then used to eliminate any additional EOG artefacts, muscular artifacts such as clenching, and bad electrodes. During this process, trials rejected by the semi-automated process where reviewed and were restored if the tool had been too sensitive. Remaining electrical artifacts were then removed with a 30 Hz low pass filter. Independent Component Analysis (ICA) via the Fieldtrip function ft_componentanalysis, was then used to identify ECG artefacts, and any persisting EOG, which were then removed manually based on topography and time course using ft_rejectcomponent. Finally, the Fieldtrip function ft_channelrepair was then used to replace bad electrodes via spline-interpolation, which interpolates missing data from the surrounding electrodes.

Trial averages were then computed for the tetanised and non-tetanised recordings for each session ft_timelockgrandaverage at each of the recording times of pre-tetanus, early post-tetanus, and late post-tetanus. Individual difference waves for the early (early post-tetanus minus pre-tetanus) and late (late post-tetanus minus pre-tetanus) recordings of each session were then computed using the Fieldtrip function ft_math, before being converted from Fieldtrip to SPM format using spm_eeg_ft2spm.

In preparation for analysis files were converted to SPM12 format. These trial averages were then smoothed with a 6 x 6 x 6 x FWHM Gaussian kernel and converted into NIfTI images for SPM analysis with a 0 – 250ms time window. Based on previous LTP research (3, 4), an occipital-parietal region of interest (ROI) was defined, consisting of electrodes P1, P2, P3, P4, P5, P6, P7, P8, Pz, PO3, PO4, PO7, PO8, PO9, PO10, POz, O1, O2 and Oz.

Parameter-Finding Planned Contrasts

Initial parameter-finding analysis for each of the Baseline vs Treatment and Baseline vs Final analyses were conducted on the difference waves using SPM12 with a 2 x 2 ANOVA of Time (Early/Late) x Stimulus (tetanised/non-tetanised), with planned contrasts outlined in Table S4. Specific peaks of interest were lateralised negativity in the early condition representing changes in the N1b component, and central positivity in the Late condition, representing changes in the P2 component. Planned contrasts were one-tailed t-tests and effects were considered significant if the Familywise Error Corrected (FWE-c) p-value was < 0.05. Specificity was identified if difference waves following the tetanised stimulus difference from those of the non-tetanised stimulus.

Table S4: Planned contrasts for parameter finding in the LTP task for the factors Time (Early/Late) and Simulus (Tetanised/Non-tetanised)

Target	Analysis	Direction	Threshold	Location	Contrast
Early potentiation	One sample one-tailed *t*-test	Negative	FWE-c <0.05	Lateralised	-1 -1 0 0
Late potentiation	One sample one-tailed *t*-test	Positive	FWE-c <0.05	Central	0 0 1 1
Early specificity	Two sample two-tailed *t*-test	Any	FWE-c <0.05	Any	-1 1 0 0

Late specificity	Two sample two-tailed *t*-test	Any	FWE-c <0.05	Any	0 0 1 -1

Source Analysis

Source analysis followed methods previously reported by our lab (3, 5). Group inverse reconstruction analysis was undertaken using Multiple Sparce Priors in SPM12 (6). A standard 64-channel actiCAP template of electrode locations with standardise fiducials was used, with SPM's inbuilt MNI structural template used in the absence of individual structural MRI scans. A time window of 160-250ms was used to encompass the time windows of both the early and late analyses. Statistical analysis was a 2 × 2 repeated measures ANOVA was run ([early post-tetanus vs late post-tetanus for the baseline session] x [LSD vs Placebo]). A peak voxel was selected from the most intense *F* value for contrast that encompassed both early and late effects of the photic tetanus for the LSD and Placebo groups. The selected peak was extracted from each participant as the local field potential (LFP), with a 5-mm spherical radius around the MNI coordinate MNI = [-10 -98 -8] in the left calcarine cortex.

Thalamo-cortical Model

Dynamical causal modelling (DCM) employed a thalamo-cortical model (7) previously applied to LTP data by our group (3, 5). While the standard DCM architecture (cmm_NDMA) models four populations of cortical cells (layer II/III superficial pyramidal (SP); layer IV spiny stellate (SS); layer V deep pyramidal (DP); and a combined pool inhibitory interneurons), the thalamocortical model used here expands this to include two thalamic nodes (excitatory reticular (RT) and inhibitory relay cells (RL)), as well as separate populations of deep (layer V; DI) and superficial (layer II/III; SI) inhibitory interneurons, and layer VI pyramidal cells with projections to the thalamus (TP) (Figure 2). This model differentiates separate AMPA, NMDA, $GABA_A$, $GABA_B$, m-current, and h-current channels.

Following the Douglas and Martin canonical microcircuit model (8), and in line with previous uses of this model (3, 5, 7) the parameters allowed to vary were RL➔ SS, SS ➔SS, SS ➔ SP, SS ➔ SI, SI ➔ SP, SI ➔ SI, SP ➔ SP, SP ➔ SI, SI ➔ SS, SP ➔ DP, DP ➔ TP, TP ➔ RL, and the rest of the parameters were fixed.

Parametric Empirical Bayes

Analysis of the parameter differences was conducted using Parametric Empirical Bayes (PEB) (9) in a 'PEB of PEBs' method (10). Bayesian model reduction was used to iteratively search all possible parameter contributions to the effect of interest, reducing free parameters until only those which meaningfully contribute to the model evidence remain. First, this was implemented as a first order PEB conducted on the average effect (a column of 1s) and a within-subjects contrast of the Baseline vs

Treatment and Baseline vs Final sessions separately (a column of Baseline as -1s and Treatment/Final as 1s). The re-estimated DCMs were entered into the second PEB on the average (column of 1s) and this time the between subjects contrast of the LSD vs placebo groups (a column of Placebo as -1s and LSD as 1s). Bayesian model averaging was implemented to identify the direction and size of the effect of this final subset of parameters on the effect of LSD versus Placebo. A threshold of 'very strong' evidence was applied to the free energy parameter estimates (posterior probability > 0.99).

Additional Results

Demographics

Table S5: Demographics of all randomised participants by treatment group (N = 80)

		Baseline-Treatment		Baseline-Final	
**Observation**		**Placebo**	**LSD**	**Placebo**	**LSD**
Age, *M* (*sd*)		36.6 (7.2)	37.8 (9.5)	37.4 (7.3)	37.5 (9.2)
Weight, kg, *M* (*sd*)		84.3 (14)	86.1 (15)	84.4 (13.1)	86.4 916.2)
BMI, kg/m^2, *M* (*sd*)		26.7 (2)	26.7 (1.8)	25.9 (2)	27.1 (1.9)
Lifetime serotonergic psychedelic use, Mdn (IQR)		2.5 (0.75-7.250	3.0 (0-10)	1 (0-7)	3 (0-10)
Psychedelic naïve, *n* (%)		9 (25)	11 (29.7)	10 (38.5)	7 (28)
Education	No qualification, *n* (%)	1 (2.7)	0 (0)	1 (3.8)	0 (0)
	Secondary school, *n* (%)	2 (5.6)	6 (16.2)	1 (3.8)	6 (24)
	Tertiary Certificate or Diploma, *n* (%)	4 (11.1)	6 (16.2)	2 (7.7)	6 (24)
	Bachelor's Degree, *n* (%)	14 (38.9)	15 (40.5)	12 (46.2)	15 (60)
	Postgraduate Diploma/Honors *n* (%)	6 (16.7)	4 (10.8)	6 (23.1)	4 (16)
	Master's Degree, *n* (%)	7 (19.4)	7 (18.9)	3 (11.5)	7 (28)
	Doctoral Degree, *n* (%)	2 (5.6)	2 (5.4)	1 (3.8)	2 (8)
Ethnicity	Asian, *n* (%)	3 (8.3)	4 (10.8)	2 (7.7)	3 (12)
	Latin American/Caribbean, *n* (%)	3 (8.3)	0 (0)	2 (7.7)	0 (0)
	Māori, *n* (%)	2 (5.6)	1 (2.7)	2 (7.7)	0 (0)
	New Zealand European, *n* (%)	26 (72.2)	24 (64.9)	20	16 (64)
	Other European, *n* (%)	4 (11.1)	11 (29.7)	1 (3.8)	5 (20)
	Pasifika, *n* (%)	0 (0)	2 (5.4)	0 (0)	2 (8)
	Other, *n* (%)	3 (8.3)	1 (2.7)	1 (3.8)	0 (0)

Note: All participants in this study are male. Ethnicity percentages will add up to more than 100 due to multiple ethnicities reported by single participants. Self-reported ethnicities of each group are given in Murphy *et al.* (2023) (2).

Parameter-Finding Results

Parameter-finding analyses (Table S6) identified significant lateralised negative peaks in the Early conditions post-stimulus for both analyses peaking in the right hemisphere at 183ms in the Baseline vs Treatment analysis ($t_{(580)}$ = 7.55, p = < 0.0001 FWE-c) and at 180ms in the Baseline vs Final analysis ($t_{(404)}$ =6.40, p = < 0.0001 FWE-c), consistent with a potentiated N1b, with specificity identified peaking at 119ms in both analyses (Baseline v Treatment $t_{(580)}$ = 8.57, p = <0.0001 FWE-c; Baseline vs Final $t_{(404)}$ = 8.94, p < 0.0001 FWE-c). A significant positive component was identified in both analyses in the Late condition, with peaks at 181ms in the Baseline vs Treatment analysis ($t_{(580)}$ = 25.2, p < 0.0001 FWE-c) and 179ms in the Baseline vs Final analysis ($t_{(404)}$ = 20.43, p < 0.0001 FWE-c), with subpeaks occurring from 179-240ms, consistent with potentiated P2, with no specificity identified in any Late analysis (Table S3). This is consistent with previous implementation of the paradigm (4). As such, time windows for analysis were determined to be 160-200ms in the Early condition with only tetanised stimuli included in analysis, and 170-250 in the Late condition with both tetanised and non-tetanised stimuli averaged. Raw ERPs for each analysis are given in Figures S1 and S2.

Table S6: Parameter finding results for the LTP task.

Contrast	Location	t	df	FWE-c *p*	Time	Specificity
Baseline vs Treatment						
Early negative lateralized potentiation	Left	6.2	580	<0.001	179	78 & 119 ms
	Right	7.55	580	<0.001	183	
Late positive central potentiation	Central	25.2	580	<0.001	181	Nil
	Central	15.51	580	<0.001	237	
	Central	15.5	580	<0.001	220	
Baseline vs Final						
Early negative lateralized potentiation	Right	6.4	404	<0.001	180	119ms
	Left	5.44	404	<0.001	178	
Late positive central potentiation	Central	20.43	404	<0.001	179	Nil
	Central	15.04	404	<0.001	240	
	Central	14.73	404	<0.001	238	

VEP Components

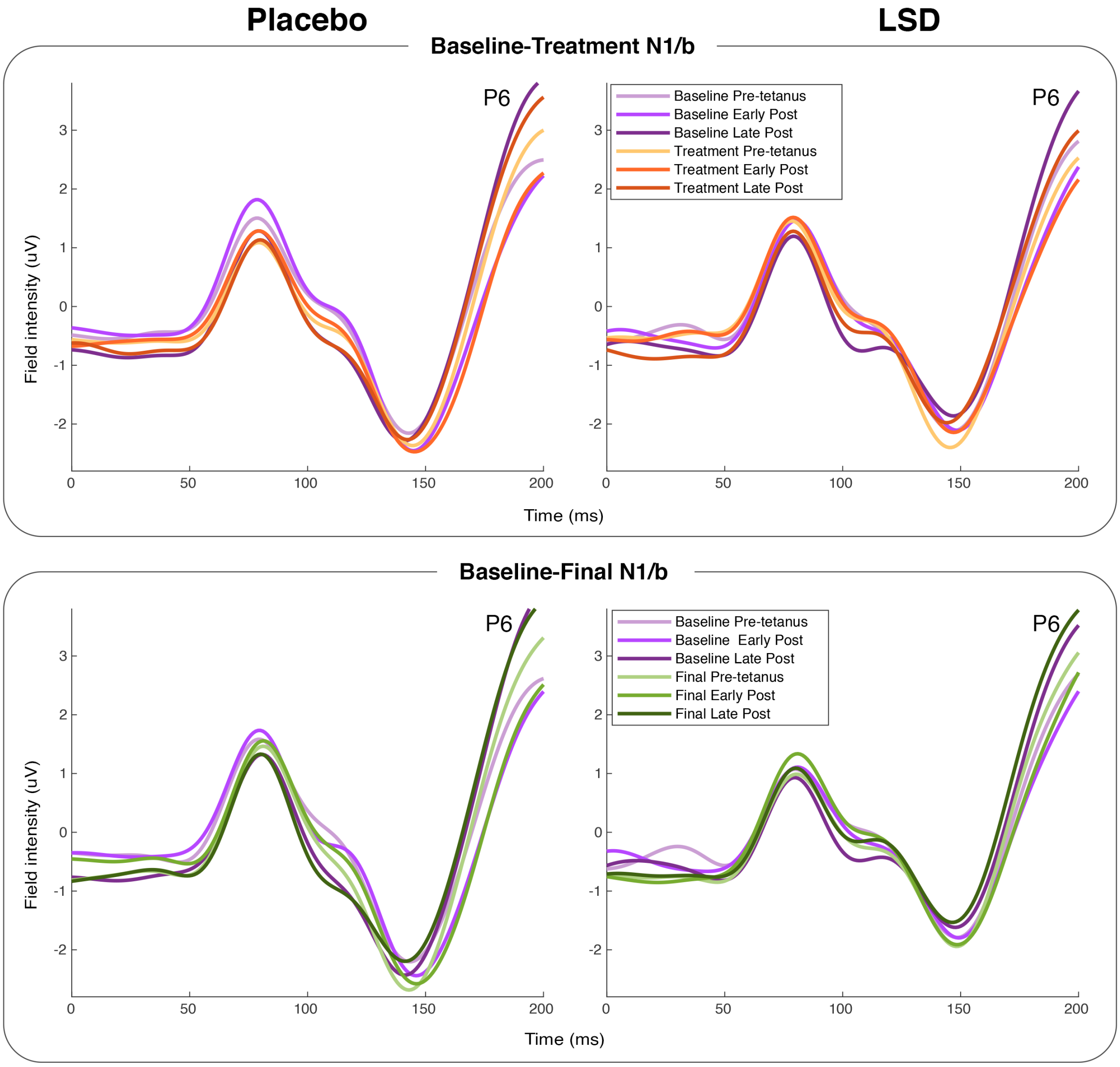

Figure S1: VEP of N1/b component as illustrated at electrode P6 for Placebo and LSD groups in the Baseline vs Treatment and Baseline vs Final analyses. P6 here is illustrative only, analysis was conducted on a 19 electrode occipital-parietal ROI.

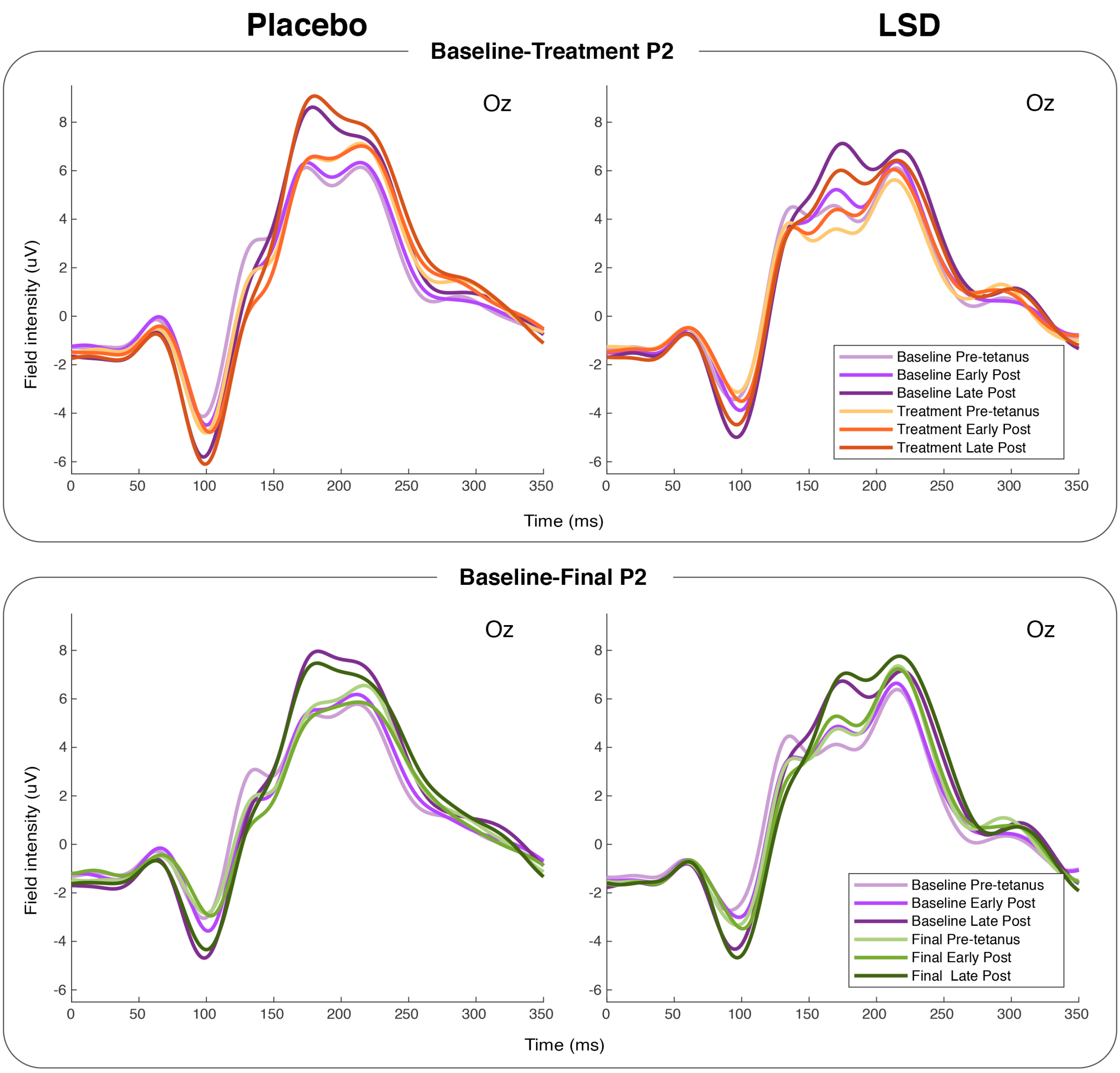

Figure S2: VEP of P2 component as illustrated at electrode Oz for Placebo and LSD groups in the Baseline vs Treatment and Baseline vs Final analyses. Oz here is illustrative only, analysis was conducted on a 19 electrode occipital-parietal ROI.

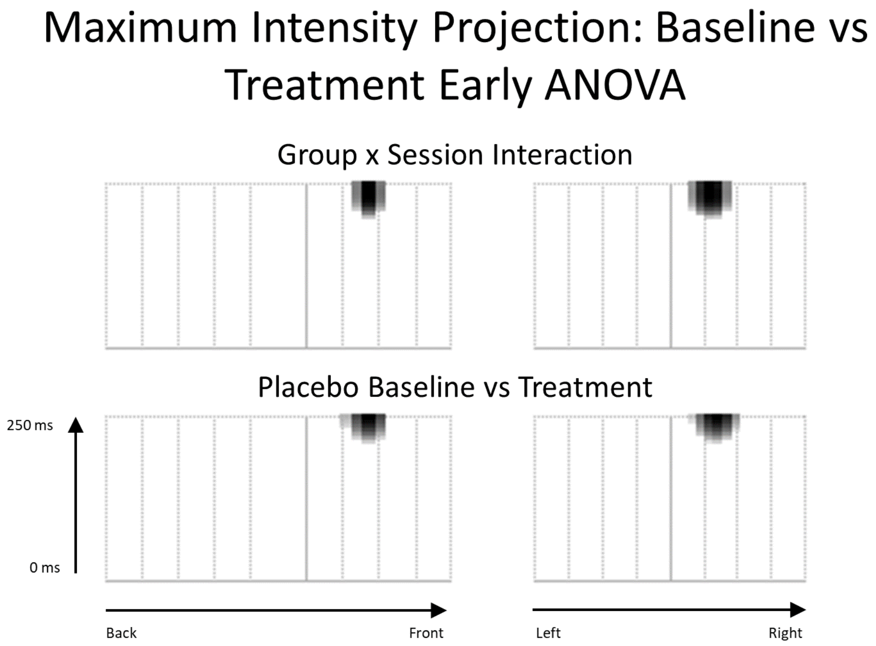

Figure S3: Maximum Intensity Projection (MIP) images from SPM12 results of Baseline vs Treatment analysis showing Group x Time interaction, main effect of Group, and post-hoc contrast of Baseline vs Treatment in the Placebo group. Note: Standard glass brain MIP not shown as occipital-parietal selection of electrodes is not rendered accurately on the glass brain.

Table S7: Parameter estimates of difference in LSD group relative to placebo in the Baseline to Treatment and Baseline to Final visits. Positive values indicate greater change in the LSD group, negative values indicate greater change in the placebo group.

	**Treatment**			**Final**		
	**Ep**	**sd**	**Pp**	**Ep**	**sd**	**Pp**
**SS -> SS**	-0.01679	0.008887	0.99	-1.93E-06	0.000236	
**SS -> SP**	0.01005	0.007849		3.83E-06	0.000235	
**SS -> SI**	-0.03392	0.02936		-3.50E-06	0.000242	
**SP -> SP**	0.04488	0.02053	1.00	-6.54E-07	0.00023	
**SP -> SI**	1.64E-06	0.000218		-9.46E-08	0.000243	
**SP -> DP**	-0.01838	0.009018	0.99	-0.02079	0.01009	1.00
**SI -> SS**	0.09366	0.04949	1.00	0.1292	0.05601	1.00
**SI -> SP**	0.09515	0.03693	1.00	5.37E-06	0.00023	
**SI -> SI**	0.02565	0.05477		-4.81E-06	0.000239	
**DP -> TP**	-8.16E-06	0.000225		-0.01161	0.009098	
**TP -> RL**	3.30E-06	0.000234		0.009762	0.01823	
**RL -> SS**	-8.11E-07	0.000229		-0.00436	0.01582	
**AMPA**	0.01672	0.008155	0.99	0.01639	0.01056	
**NMDA**	-0.015	0.006545	0.99	-0.01578	0.007548	1.00

References

1. Murphy RJ, Sumner RL, Evans W, Menkes D, Lambrecht I, Ponton R, et al. MDLSD: study protocol for a randomised, double-masked, placebo-controlled trial of repeated microdoses of LSD in healthy volunteers. Trials. 2021;22(1):1-15.

2. Murphy RJ, Sumner R, Evans W, Ponton R, Ram S, Godfrey K, et al. Acute mood-elevating properties of microdosed LSD in healthy volunteers: a home-administered randomised controlled trial. Biological Psychiatry. 2023;94(6):511-21.

3. Stone E, Alshakhouri M, Shaw A, Muthukumaraswamy S, Sumner RL. Changes in Visual Long-term Potentiation Show Preserved Cyclicity in Human Females Taking Combined Oral Contraceptives. Neuroendocrinology. 2023:1-.

4. Sumner RL, McMillan R, Spriggs MJ, Campbell D, Malpas G, Maxwell E, et al. Ketamine enhances visual sensory evoked potential long-term potentiation in patients with major depressive disorder. Biological Psychiatry: Cognitive Neuroscience and Neuroimaging. 2020;5(1):45-55.

5. Sumner RL, Spriggs MJ, Shaw AD. Modelling thalamocortical circuitry shows that visually induced LTP changes laminar connectivity in human visual cortex. PLoS Computational Biology. 2021;17(1):e1008414.

6. Litvak V, Friston K. Electromagnetic source reconstruction for group studies. Neuroimage. 2008;42(4):1490-8.

7. Shaw AD, Muthukumaraswamy SD, Saxena N, Sumner RL, Adams NE, Moran RJ, et al. Generative modelling of the thalamo-cortical circuit mechanisms underlying the neurophysiological effects of ketamine. NeuroImage. 2020;221:117189.

8. Douglas RJ, Martin KA. Neuronal circuits of the neocortex. Annu Rev Neurosci. 2004;27:419-51.

9. Friston KJ, Litvak V, Oswal A, Razi A, Stephan KE, Van Wijk BC, et al. Bayesian model reduction and empirical Bayes for group (DCM) studies. Neuroimage. 2016;128:413-31.

10. Casey CP, Tanabe S, Farahbakhsh Z, Parker M, Bo A, White M, et al. Dynamic causal modelling of auditory surprise during disconnected consciousness: the role of feedback connectivity. Neuroimage. 2022;263:119657.
